# Subject-specific, multiscale simulation of electrophysiology: a software pipeline for image-based models and application examples

**DOI:** 10.1098/rsta.2008.0314

**Published:** 2009-06-13

**Authors:** R.S. MacLeod, J.G. Stinstra, S. Lew, R.T. Whitaker, D.J. Swenson, M.J. Cole, J. Krüger, D.H. Brooks, C.R. Johnson

**Affiliations:** 1 Nora Eccles Harrison Cardiovascular Research and Training Institute (CVRTI), University of UtahSalt Lake City, UT 84112, USA; 2 Scientific Computing and Imaging (SCI) Institute, University of UtahSalt Lake City, UT 84112, USA; 3 Department of Bioengineering, University of UtahSalt Lake City, UT 84112, USA; 4 Center for Communications and Digital Signal Processing (CDSP), Northeastern UniversityBoston, MA 02115, USA

**Keywords:** simulation, geometric modelling, mesh generation, electrophysiology, defibrillation

## Abstract

Many simulation studies in biomedicine are based on a similar sequence of processing steps, starting from images and running through geometric model generation, assignment of tissue properties, numerical simulation and visualization of the results—a process known as image-based geometric modelling and simulation. We present an overview of software systems for implementing such a sequence both within highly integrated problem-solving environments and in the form of loosely integrated pipelines. Loose integration in this case indicates that individual programs function largely independently but communicate through files of a common format and support simple scripting, so as to automate multiple executions wherever possible. We then describe three specific applications of such pipelines to translational biomedical research in electrophysiology.

## 1. Introduction

Many simulation studies in biomedicine are based on a similar sequence that starts from images and runs through geometric model generation, assigning tissue properties, numerical simulation and visualization of the results. The images, often sets of images that combine to describe volumes, come from many modalities and the task then becomes to identify structures of interest and describe those structures in a form suitable for the numerical solution of equations that describe the function of these structures. Thus, one can define a pipeline for image-based model generation and simulation that, once created, can find use in many different fields of biomedical (or other) science and engineering. The desire for such models has, in turn, created a pressing need for software tools that extract anatomy and tissue characteristics from the images, create computational meshes and allow assignment of relevant parameters to the resulting geometric model.

Unfortunately, another trait common to many areas of biomedical simulation is the lack of available software, especially those in the public domain, to carry out all the steps of this pipeline. A major goal of our research and development is to address this need and we have created a set of software tools that support simulation pipelines in at least a few application domains. Wherever possible, we have maintained a high level of generality in the software and the algorithms they combine; however, we propose that, in many situations, there are substantial benefits to adapting software to a particular application that outweighs the resulting inevitable loss of generality. Moreover, by striking a suitable balance between the generality of the simulation pipeline and the specific requirements of a problem domain, our experience suggests that one can achieve another major objective of contemporary biomedical simulation, which is creating subject-specific implementations of clinically relevant numerical simulations. We will describe examples of subject-specific models that we have developed, as well as highlight a few of the outstanding challenges that arise in this setting.

### (a) Image-based modelling

Interest in image-based modelling is based on the growing access of biomedical scientists to three- (and even four-) dimensional imaging that allows the creation of simulation models that, in some cases, include explicit and individualized anatomical information from the objects under study. Where previous models have used highly simplified representations of biological tissues based on simple shapes (lines, sheets, spheres, cylinders, etc.), it is now possible to acquire sets of images of all manner of cells, tissues and organs using modalities such as microscopy (e.g. histological serial sections, electron tomographic or confocal), X-rays (e.g. biplanar fluoroscopy or computed tomography (CT)), nuclear medicine (e.g. single photon emission computed tomography (SPECT), positron emission tomography (PET)), magnetic resonance (anatomical, T1, T2 or diffusion weighted) or ultrasound. These images then provide a means to create models that are highly realistic and even subject- (or patient-) specific in their anatomical or geometric aspects.

**Figure 1:**
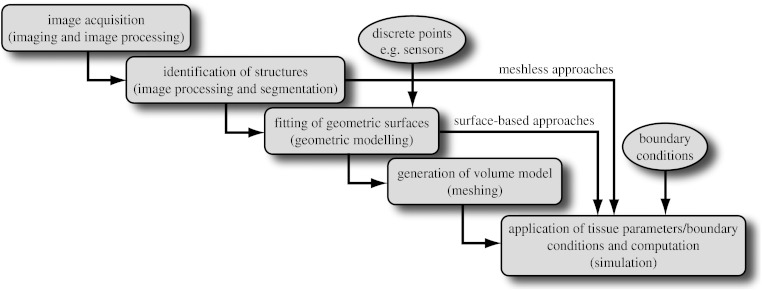
Schematic of a simulation pipeline. Each element has a functional title and then, in parentheses, the technical description of the associated task.

Figure 1 shows a diagram of the resulting workflow that applies to many problems in biomedical simulation and contains the following elements:

- image acquisition and processing for a tissue, organ or region of interest (imaging and image processing),
- identification of structures, tissues, cells or organelles within the images (image processing and segmentation),
- fitting of geometric surfaces to the boundaries between structures and regions (geometric modelling),
- generation of three-dimensional volume mesh from hexahedra or tetrahedra (meshing), and
- application of tissue parameters and boundary conditions and computation of spatial distribution of scalar, vector or tensor quantities of interest (simulation).

Naturally, there are variations possible at each step in the pipeline depending on, for example, the imaging modalities that are relevant and available, the physical scale of the problem domain or the mathematical equations and their numerical approximations. The arrows in figure 1 show an example of the output of surface fitting becoming the basis of application of boundary conditions and simulation.

### (b) Software infrastructure

There are different approaches to addressing any multifaceted software project and the choice of the structure of the software is critical to the success of the project. The need for careful design is especially critical when the software is to serve a diverse community of domain-specific experts. Decisive factors include ease of use, flexibility to adapt to a wide range of data sources and applications, robustness, efficiency and support for multiple platforms. It is also desirable to have a certain degree of integration among different aspects of the software so that users see a similar interface and use familiar terms across each step in the workflow. An additional factor in the design of a large-scale software system is whether to employ a ‘top-down’ design or to iterate from specific to general solutions through a ‘bottom-up’ design that starts with elemental solutions tailored to specific applications and then seeks to integrate them.

There are numerous open-source software systems for biomedical simulation and one can organize them according to their degree of coverage from both their technical capabilities and the breadth of their application domain. One category of such software achieves broad technical coverage across a very general application domain. Others, by contrast, target a particular application domain and provide comprehensive and/or integrated solutions within that domain. A third category includes programs that are very specific in their technical coverage but are generalized in terms of the application domain. The final category is the programs that are focused in terms of both technical capacity and breadth of application. The list of open-source examples below, organized by these criteria, is meant to be representative and not comprehensive, and we apologize for inevitable omissions. We have limited the list to our first-hand knowledge, thus have also omitted commercial software.

#### (i) Comprehensive technical and broad application domain

These are the truly comprehensive systems that include a wide range of technical tools that can be combined into workflows and applied to a wide range of specific biomedical (or even more broadly scoped) problems.

- SCIRun (software.sci.utah.edu/scirun) is our own example of a general purpose, problem-solving environment that has found extremely broad application both within biomedicine (Johnson *et al*. 2002; Henriquez *et al*. 2004; Stinstra *et al*. 2005; Wolters *et al*. 2006; Jolley *et al*. 2008) and in areas as diverse as nuclear physics (Sanderson *et al*. 2004; Jones *et al*. 2007) and combustion (Parker 2006).
- CMISS (www.cmiss.org) also has a very broad technical scope and application domain (Blackett *et al*. 2005), and is the basis of many simulation studies in bioelectric fields and biomechanics of the heart and other organs (Hooks *et al*. 2002; Garny *et al*. 2003; Nash 2005), respiratory physiology (Tawhai *et al*. 2000) and bioelectric fields in the gastrointestinal system (Pullan *et al*. 2004).
- Simbios (simbios.stanford.edu) is a newly emerging software system from the NIH-funded Center for Physics-based Simulation of Biological Structures (Schmidt *et al*. 2008). The biological coverage of Simbios is very broad, with the goal to help biomedical researchers understand biological form and function as they create novel drugs, synthetic tissues, medical devices and surgical interventions (Blemker *et al*. 2007; Delp *et al*. 2007; Besier *et al*. 2008; Bowman *et al*. 2008).
- 3D Slicer (www.slicer.org) is a multi-platform, open-source set of tools for visualization and image computing. It is also from an NIH NCBC Center, the National Alliance for Medical Image Computing (NA-MIC; www.na-mic.org; Pieper *et al*. 2006). Slicer includes a wide variety of image processing and visualization capabilities, including segmentation, registration and analysis (Lankton & Tannenbaum 2008; Maddah *et al*. 2008).

#### (ii) Comprehensive technical with focused application

Software systems that provide comprehensive technical support for a specific application area in biomedicine.

- Brainstorm (neuroimage.usc.edu/brainstorm) is an integrated toolkit dedicated to visualization and processing of data recorded from magnetoencephalography (MEG) and electroencephalography (EEG). Brainstorm provides a comprehensive set of tools for researchers interested in MEG/EEG (N'Diaye *et al*. 2004; Sergent *et al*. 2005; Jerbi *et al*. 2007).
- SimBio and NeuroFEM (www.simbio.de and www.neurofem.com) are a combination of programs directed at source localization in the brain using patient-specific finite-element models with multiple conductivities and even anisotropic conductivity (Wolters *et al*. 2006).
- Continuity (www.continuity.ucsd.edu) is a problem-solving environment for multiscale modelling in bioengineering and physiology with special emphasis on cardiac biomechanics, transport and electrophysiology.
- PCEnv (www.cellml.org/downloads/pcenv) is the Physiome CellML Environment, an integrated software that provides an interface to the cell simulation models of the CellML project.
- Virtual Cell (www.nrcam.uchc.edu) is a software system for a wide range of scientists, from experimental cell biologists to theoretical biophysicists, who wish to create models of cellular structure and chemical, electrical or mechanical function.
- Neuron (www.neuron.yale.edu/neuron) is a simulation environment for modelling individual neurons and networks of neurons, which is especially well suited to comparisons with experimental data. It has a very user-friendly interface that provides tools for building, managing and using models in a way that is numerically sound and computationally efficient.
- Genesis (www.genesis-sim.org) has a very similar application domain to Neuron as a general purpose simulation platform to simulate neural systems ranging from subcellular organelles and biochemical reactions to complex models of single neurons, large networks and system-level models.

#### (iii) Focused technical and broad application domain

Software systems that solve a technical need very well and become the basis for integrated systems in a wide range of application areas.

- TetGen (tetgen.berlios.de) creates tetrahedral volume meshes from volume data made from triangulated surfaces for solving partial differential equations by finite-element or finite-volume methods. TetGen is an integrated component in some of the modelling pipelines described here.
- Insight toolkit (ITK, www.itk.org) is a comprehensive set of software functions to perform image processing or analysis. ITK is the basis of many other tools (e.g. SCIRun and Seg3D) as they lack a graphical user interface (GUI) and exist only as a C++ class library (Ibanez & Schroeder 2005).
- The Visualization toolkit (VTK, www.vtk.org), which consists of an extensive library for visualization functions, is a component in many larger systems, e.g. 3D Slicer (Schroeder *et al*. 2006).

#### (iv) Focused technical and focused application domain

There are a number of highly successful systems that have a highly focused set of capabilities and applications.

- ECGSim (www.ecgsim.org) is a program that computes the body surface potentials from the heart and allows the user to make changes in the electrical characteristics of the cells in any region of the heart. Its goal is not only to provide an educational tool but also a way to study the relationship between the electrical activity of the ventricular myocardium and the resulting potentials on the thorax under both normal and pathological conditions.
- LabHeart (www.labheart.org) is primarily a teaching tool that simulates the cardiac action potential, including the individual ionic currents and the fluctuations in intracellular calcium concentration.
- iCell is an internet-based simulation program that allows the user to generate action potentials from a wide range of cell types (Demir 2006).

These examples include both bottom-up and top-down solutions. The larger, more comprehensive and general purpose software systems tend to be top-down as they are integrated from the conception, while the more focused systems have become components of loosely coupled bottom-up implementations.

## 2. Methods and implementations

We describe here a collection of methods that make up the elements of the image-based modelling and simulation pipeline illustrated in figure 1 and how we have implemented such pipelines. The technical level of the description will be modest and we defer throughout to other more detailed reports.

### (a) Software pipeline and infrastructure

One goal in scientific software is to create suites of relatively general purpose, ideally open-source tools that are modularized, so that it is easy to replace any particular step and maintain all the benefits of the remaining elements. The flexibility afforded by a modular approach is essential because of the always changing needs of the application scientist and also because of the advantages of a distributed development process. In such an environment, programs are the product of teams of professional programmers, students, postdoctoral fellows or investigators, with highly variable levels of coordination among those submitting changes to the code. The higher the level of autonomy among elements of the system and the simpler the means that the elements communicate, the less coordination and agreement there must be among the development team and the lower the cost to the whole system of changes made in any one module. The advantages of open-source development have been well documented (O'Reilly 1999) and recent changes in public funding policy have provided further motivation for making software developed by public funding available to the academic community.

We have considerable experience with both the top-down and bottom-up approaches to software architecture (MacLeod & Johnson 1993; Johnson *et al*. 2002, 2004; [Bibr bib7][Bibr bib8][Bibr bib9][Bibr bib10]; SCI Institute 2008*a*,*b*) and have found advantages and disadvantages to each. The top-down approach ensures tight coordination and integration of software components; data storage and structures are common across modules, application programming interfaces (APIs) between modules are standardized and GUIs can maintain a consistent appearance and terminology, which can reduce the time required for users to learn to use the resulting software. However, top-down design requires prescient appreciation of all the potential uses of the software and a keen sense of anticipation of not only the immediate goals but also the future applications. Top-down systems also seek to abstract the operations and interactions, to find generic terms for steps that may have domain-specific names, thus potentially challenging the new user and slowing down the learning process. The advantages of the bottom-up approach are the complements; programs arise and evolve in direct response to the applications and are thus very well tuned to the workflow and the nomenclature of the field. They are often smaller with less elaborate internal architecture and thus can be easier to write, often the product of a single person or a very small team.

Our approach has been something of a hybrid or even a parallel system. On the one hand, we continue to use and expand SCIRun, a very general purpose problem-solving environment, to test algorithms and approaches and develop application-specific solutions. SCIRun is a stable platform that exhibits many of the advantages of the top-down design strategy—data structures and APIs are consistent and predictable and there is a high degree of code sharing and usefulness of capabilities developed previously. Using SCIRun greatly simplifies interactive visualization and steering of the process because of pre-existing capabilities and provides extensive support for the simulation component of the project. However, the interface to SCIRun is complex for a biological user and even the nomenclature of the user interface elements is quite computer technical rather than biomedical, further intimidating the typical biomedical user. As a result, the burden of setting up and carrying out the simulations often falls more to the technical members of the team than to the biomedical collaborator.

We have also followed the complementary path of developing a small, focused and standalone software system for tasks that are ubiquitous across many projects. Our oldest example of this strategy is Map3d, which is an interactive visualization program created for multichannel time signals whose spatial organization is in surfaces (MacLeod *et al*. 1992; CIBC 2008*e*). The program has a highly focused set of technical capabilities and the application domain has traditionally been cardiac or neural electrophysiology (Punske *et al*. 2005; Ciaccio *et al*. 2008). The program is also the creation of a very small team, one of whom is a biomedical scientist so that the interface and the nomenclature are familiar and easily adopted by the target community.

### (b) Implementation of the pipeline

#### (i) Top-down approach

We have implemented within SCIRun several meshing and mesh refinement schemes based on hexahedral and tetrahedral elements and used them extensively in the example of simulation of cardiac defibrillation described in detail in §3. Most of these meshing schemes start by overlaying a regular grid on top of the voxelized images and then turning them into a model by adding local refinements and boundaries based on the needs of the simulation. Although carrying out refinement to an existing mesh is a relatively straightforward task, it becomes much more challenging when maintaining good mesh quality, i.e. controlling the shape and size of the elements. In biological problems, meshes often require embedding of irregularly shaped boundaries of different tissue properties as well as adding local refinements for detailed simulations around biological sources. We have developed novel methods to approximate such features using hexahedral meshes that also allow the addition of irregular boundaries while still maintaining high mesh quality.

#### (ii) Bottom-up approach

**Figure 2:**
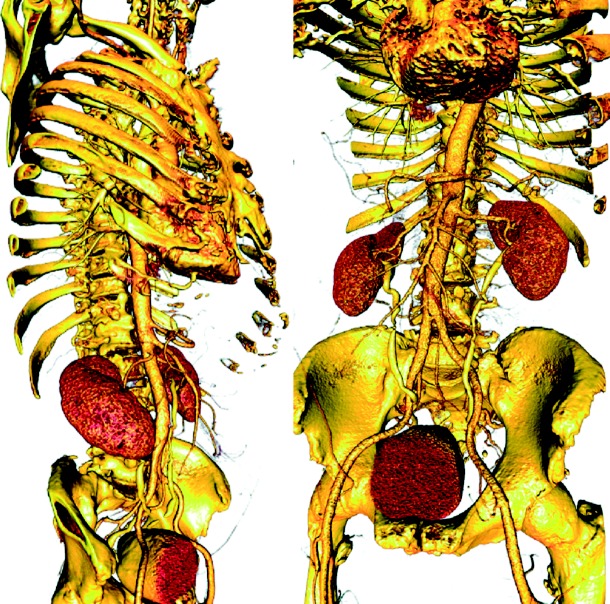
Example of volume rendering with ImageVis3D of a torso model based on a high-resolution CT scan (512×512×3172 with a voxel size of 0.51×0.51×0.50 mm, courtesy of Siemens Corporate Research, Princeton). By controlling transfer functions, it is possible to identify different systems (e.g. skeleton, vasculature) and organs (e.g. heart, kidneys and bladder).

The key to benefitting from the bottom-up approach is creating efficient and flexible elemental pieces that can interact through simple passing of data via files. Pipeline structures, in general, lend themselves well to this concept and we have implemented image-based modelling pipelines from such elements. Elements of this strategy include ImageVis3D and Seg3D (www.seg3d.org), which provide volume rendering and segmentation capabilities, respectively. ImageVis3D is based on our own volume rendering capabilities (figure 2), and Seg3D uses tools from the ITK (Ibanez & Schroeder 2005) and has a relatively focused technical breadth. Seg3D reads stacks of images as a volume using standard file formats and provides a set of tools to identify different regions within the image volume and thus generate a ‘label map’ of the volume. The nomenclature of both ImageVis3D and Seg3D is largely generic and not specific to any particular application domain and both are small programs, created within a year by a small team with the goal of facilitating rapid addition of new features or adjustments to the user interface. While these are separate programs, they integrate functionally into the workflow through files, which they can flexibly read and write.

In some applications, we have also used a second segmentation tool, 3D Slicer (www.slicer.org), which is part of the NA-MIC kit (see §1). Although Slicer is much more than a segmentation program, it is also portable and flexible enough to serve as a dedicated segmentation tool in the simulation pipeline. Integration occurs, as with Seg3D, by means of compatible file formats using the Near Raw Raster Data (NRRD) format and the associated TEEM toolkit for accessing and writing NRRD files.

Another component of all our tetrahedral mesh generation pipelines is TetGen (tetgen.berlios.de), at the moment the most effective and robust open-source tetrahedral mesh generation program, especially for cases with multiple embedded surfaces. It is this ability to deal effectively with the internal boundaries between regions of different characteristics (e.g. electrical conductivity or optical opacity) and to maintain the integrity of outer boundaries, especially when they are concave, which are essential and challenging requirements for applications in biomedicine.

In a recent description, we have outlined the details of a new mesh generation, BioMesh3D, which includes support for both hexahedral and tetrahedral mesh elements (Callahan *et al*. 2009). BioMesh3D makes use of TetGen and other meshing tools and handles the integration and user interface to modular programs and libraries.

## 3. Examples of image-based modelling and simulation

The field of electrophysiology provides a rich domain for modelling and simulation, and has been the inspiration for many advances in computing and numerical methods, including projects that have led to Nobel Prizes. From the subcellular to the whole organism, the role of anatomy and spatial organization on the mechanisms of electrophysiology leads naturally to subject-specific models. We will describe three examples of subject-specific image-based models that illustrate the common elements of the pipeline in figure 1 over a range of sources of image and anatomical information.

### (a) Example 1: modelling of focal current sources in the brain

**Figure 3:**
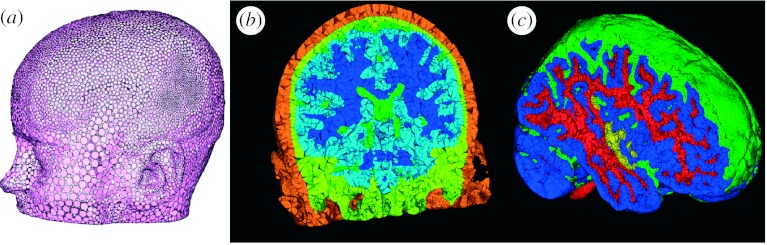
Example of meshing of the head in a paediatric epilepsy patient. (*a*) The particle distribution over the head surface and highlight of the variation in particle size, the adaptivity of the particles over the skin. (*b*) The associated tetrahedral mesh and (*c*) another higher resolution view of the mesh highlighting the cortex and cerebrospinal fluid.

Figure 3 contains geometric model results from a 15-year-old paediatric patient suffering from epileptic seizures. The segmentations came from a semi-automated tissue classification algorithm developed by Wu *et al*. (2006), followed by extensive manual inspection and hand editing of mislabelled pixels using Seg3D. The meshing component of the pipeline was implemented in BioMesh3D, a new program that incorporates separate surface-fitting and mesh generation programs (e.g. TetGen) in a scripting environment (Callahan *et al*. 2009). The triangle mesh quality in figure 3*a* is excellent, a result of the distributed particle method that we have developed (Meyer *et al*. 2005, 2007). The tetrahedral quality, as measured by radius ratio (which is indicative of the conditioning of the finite-element stiffness matrix in the resulting linear system), is not as good as for the surfaces, but still highly suitable for simulations. The most disruptive tetrahedra are slivers, a frequent product of the triangularization algorithms implemented in TetGen. One of the potential advantages of incorporating programs such as TetGen into integrated systems is a simplification for the user by optimizing, or at least limiting the range of, control parameters. TetGen has a number of user-control settings that allow it to work in a wide range of applications; by focusing on a smaller application range, it is possible to identify settings that achieve acceptable results and expose only the essential parameters to the user interface. In BioMesh3D, we have been able to generally use the same set of TetGen parameters for all cases and required only minor manual intervention after the initial tuning. Future research will focus on the computation time, which is 8–12 hours for datasets such as that in figure 3, and is mostly spent on (i) preprocessing and distributing particles, (ii) carrying out the tetrahedralization, and (iii) dealing with remnant errors in the mesh, such as slivers.

**Figure 4:**
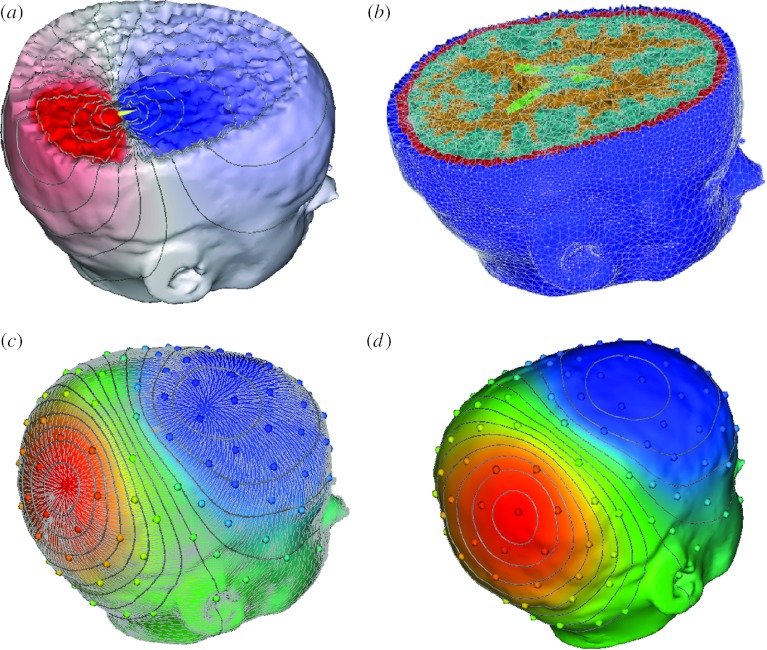
(*a–d*) Illustration of simulation of electromagnetic field propagation in a patient-specific brain model. The figure shows a finite-element method discretization of Poisson's equation with a patient-specific, five-compartment, geometrical model derived from a segmentation of brain magnetic resonance imaging. The solid lines in the simulation images indicate isopotentials and the small white lines are electrical current streamlines.

Figure 4 shows simulation results from a patient-specific model of the head carried out with NeuroFEM (for source simulation) and SCIRun (for mesh generation and visualization). The mesh was composed of 179 643 nodes and 1 067 541 tetrahedral elements and the preliminary simulation was carried out with a dipole source in the right posterior region. Future improvements here will focus mainly on the incorporation of diffusion tensors and then the inverse computation to identify bioelectric sources in patients with epilepsy.

### (b) Example 2: myocardial ischaemia and epicardial potentials

The goal of the second example was to create subject-specific models of the heart for use in simulating myocardial ischaemia, a condition in which the blood supply to the heart does not match the demand, which represents the physiological basis of a heart attack. The goal of the simulations was to mimic the results of experiments in which reduced blood flow to the heart in an animal model produced ischaemia that we measured electrically with high-resolution mapping systems (Shome & MacLeod 2007).

For this simulation, we selected the modelling approach known as the bidomain (Geselowitz & Miller 1983; Henriquez 1993), in which one pictures the intracellular and interstitial domains of the cardiac tissue as continuous and separate over the entire volume of the heart, linked only by the cell membrane, which is also approximated as being continuous throughout the volume. This approach is essentially a homogenization of the discrete structure of heart tissue in which it replaces the ensemble of individual cells, which each have their own intra- and extracellular volumes, with a continuous model of tissue-wide intra- and extracellular volumes. The continuous approximation is then suitable for a subsequent discretization into finite elements, which provide a means of computation.

To implement the bidomain solution for ischaemia requires an accurate discrete model of the heart that includes values for conductivity in both the intracellular and interstitial spaces. Because cardiac tissue is highly anisotropic in structure (Roberts *et al*. 1979), and this anisotropy has effects on the distribution of electric potentials (Franzone *et al*. 1982; Taccardi *et al*. 1994), a bidomain model should also include fibre structure information. The most frequently used geometric model of the whole dog heart with fibre structure comes from Nielsen *et al*. (1991) at the University of Auckland, and, in our initial studies, we also used this model to simulate ischaemia. Starting from the raw Auckland heart points, we created a parametric representation of the epicardial and endocardial surfaces using spherical harmonic basis functions in which we could easily vary the location and transmural extent of an ischaemia zone (Hopenfeld 2004). Within the ischaemia area, we assumed action potentials of 30 mV lower amplitude than surrounding healthy cells, and thus created an anisotropic source of ischaemia in a bidomain model of the entire canine heart (Hopenfeld *et al*. 2004).

**Figure 5:**
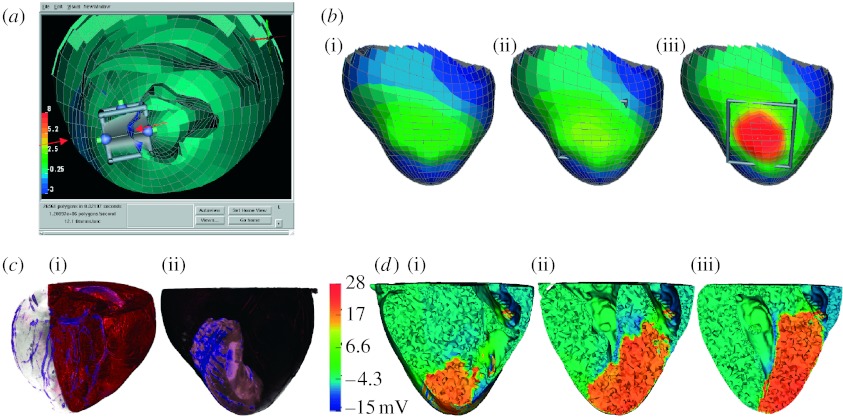
Whole-heart electrical model of ischaemia with a realistic ischaemic zone. (*a*) A single image from an interactive session using SCIRun with the three-dimensional heart geometry cut away to reveal the location of the interactive ischaemic region tool. (*b*) The associated computed epicardial potentials of a simulation of subendocardial ischaemia of progressing transmural extent ((i) 40, (ii) 70 and (iii) 90%). (*c*(i)(ii)) A volume rendering of gadolinium-enhanced images of an animal heart illustrating the coronary vessels and the perfusion bed for this heart, which we used to create subject-specific models. (*d*(i–iii)) Slices of the heart model with colour indicating the electric potential from a simulation of ischaemia in the subject-specific geometric model.

More recently, we have converted the code of the original ischaemia simulations into SCIRun modules and also performed diffusion tensor magnetic resonance imaging (MRI) of hearts from animal experiments using *post-mortem* scans on a dedicated, 7 T, small animal MRI system. Subject-specific segmentation on each heart was performed within a few hours using Seg3D and then modules in SCIRun performed all additional steps, including alignment, preprocessing of fibre orientations, meshing of the myocardium, assigning the ischaemic zone interactively and solving the potentials. Figure 5*c*,*d* illustrates initial results from these studies, including volume renderings of the coronary circulation and perfusion bed of the individual hearts.

### (c) Example 3: simulation of implantable cardiac defibrillators

The goal of these simulations was to calculate the electric potentials in the body, and especially in the fibrillating heart, which arise during a shock from an implantable cardiac defibrillator (ICD), over 90 000 of which are implanted annually in the USA alone. Of special interest was the use of such devices in children, who are both much smaller in size than adults and almost uniformly have some form of anatomical abnormality that makes patient-specific modelling essential.

We have developed a complete pipeline for the patient-specific simulation of defibrillation fields from ICDs, starting from CT or MRI image volumes and creating hexahedral meshes of the entire torso with heterogeneous mesh density in order to achieve acceptable computation times (Jolley *et al*. 2008). In these simulations, there was effectively a second modelling pipeline that was executed each time the user selected a candidate set of locations for the device and the associated shock electrodes. For each such configuration, there was a customized version of the volume mesh that had to be generated and prepared for computation.

**Figure 6:**
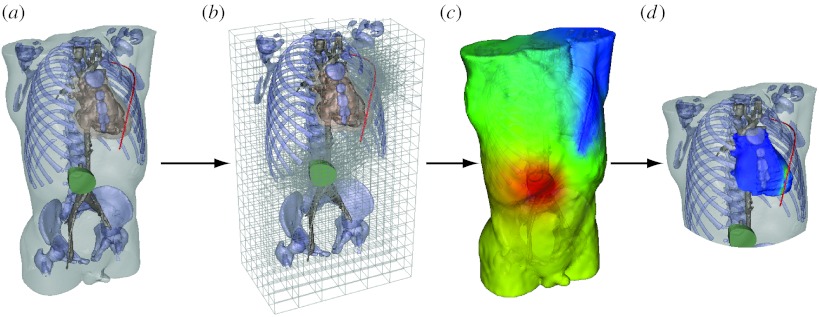
Pipeline for computing defibrillation potentials in children. The figures shows the steps ((*a*) setting electrode configuration, (*b*) refinement of hexahedral mesh for electrode locations, (*c*) finite-element solution of potentials and (*d*) analysis of potentials at the heart to predict defibrillation effectiveness) required to place electrodes and then compute and visualize the resulting cardiac potentials.

Figure 6 shows the steps required to implement the customized mesh for each new set of device and electrode locations. The user manipulated an interactive program implemented in SCIRun that allowed very flexible design and placement of the components of the device, an image of which is shown in figure 6*a*. Modules in SCIRun then carried out a refinement of the underlying hexahedral mesh, so that the potentials applied by the device and electrodes were transferred with suitable spatial fidelity to the torso volume conductor (figure 6*b*). Then additional modules in SCIRun computed the resulting electric field throughout the torso and visualized the results, also showing the details of the potentials at the heart and deriving from the simulations a defibrillation threshold value (figure 6*c*,*d*). We have also carried out initial validation of the complete system by comparing computed with measured defibrillation thresholds and obtained encouraging results (Jolley *et al*. 2008).

## 4. Discussion

Our experience in developing image-based modelling and simulation software for diverse application areas suggests several points of discussion. Some are related to the strategies of software development for this problem domain; however, we begin with an evaluation of evidence that suggests that image-based modelling and patient/subject-specific modelling are both technically feasible and scientifically desirable.

A key premise of the drive to develop efficient pipelines such as the one we describe is that creating subject-specific geometric and computational models will result in improved accuracy and more useful results. At this point, the proof to support this premise is incomplete, although intuition would suggest it to be true. For example, the relative comparisons in the defibrillation study seem to show the same trends across all the different age (and size) models, suggesting that patient-specific modelling may not be needed (Triedman *et al*. 2008). On the other hand, we see substantial influence from factors such as the presence of bowel gas on the absolute values of defibrillation potential, which would argue in favour of patient-specific modelling. The problem of source localization in the brain is perhaps at the forefront of patient-specific modelling (Huiskamp *et al*. 1999; Wolters *et al*. 2006). Of particular recent interest are cases in which EEG and MEG data from a patient are supplemented by cortical surface potentials, which, in turn, mean that the skull is disrupted in a very patient-specific manner. Such cases seem to require both highly realistic and very likely patient-specific models to achieve suitable accuracy for surgical guidance (Tao *et al*. 2007).

The question of technical feasibility is more resolved, in that modern imaging combined with recent developments in the associated software suggest that image-based modelling is, indeed, highly possible, if by no means easy. Thus, a second question arises as to which general approach, what we have described as top-down versus bottom-up, will lead to the most effective solutions. We have pursued both approaches for over 10 years and continue to develop along both lines. We have, however, begun to identify settings in which one or the other seems best suited. For our in-house development and original research in either new algorithms or their application to new biomedical questions, the advantages of the top-down or integrated environment are considerable and justify the additional time required to develop the necessary knowledge. Graduate students with suitable access to knowledgeable developers and experienced scientists can now learn to use SCIRun in days and can even develop their own modules within weeks. The resulting savings in time by having a growing suite of visualization, analysis and simulation tools available within SCIRun more than make up for the learning time. By contrast, for users from other biomedical laboratories, the technical hurdles to learning an environment as complex and flexible as SCIRun can be challenging. With enough support and the availability of customized data flow networks (the equivalent to ‘programs’ in SCIRun), a dedicated and reasonably computer literate biomedical scientists or physicians can and do use SCIRun (Jolley *et al*. 2008). More often, however, such a user will appreciate the simplicity of separate programs that are each dedicated to a piece of the image-based modelling pipeline. Our experience with programs such as Map3d, Seg3D, ImageVis3D and BioTensor all illustrate the advantages of a more limited interface in which the user can relatively quickly become facile, even at the cost of some flexibility. The reasons for this finding are open to speculation but may result from the rapid gratification of learning a small piece of software and then seeing immediate use, even if for only a small piece of the entire workflow.

A second perspective that determines the cost of developing software for use by the biomedical community is the time required to develop robust and portable programs in each of these approaches. A large integrated environment such as SCIRun has a development learning curve that can be daunting, so that even experienced professional software developers can take months to achieve the knowledge required to expand, alter or maintain the system. We have recently overhauled and simplified the API to the SCIRun infrastructure and have seen dramatic improvement in the training time required to create functional modules, a less daunting entry point and one that will satisfy the needs of most who wish to develop within SCIRun. The benefits we have seen suggest that simplification should always be a major goal of a software infrastructure, even at the cost of some numerical efficiency. However, even with such simplifications, there is a certain inertia inherent in a large system; changes in data structures, memory management or user interface can ripple through the entire system and result in months of development time. Additionally, in a system such as SCIRun that implements a particular event management, some tasks will be very efficient, while others will require undue complexity purely because of the event structure. SCIRun implements data flow, i.e. each functional module accepts data, manipulates, integrates or adds to the data stream and passes the results to one or more downstream modules. Data flow is inherently linear and sequential, which can result in unwanted overhead from frequent recomputing of many interim results each time a user makes small changes in parameters. In addition, iterative approaches are not linearly sequential and map poorly to a strict data flow paradigm.

Many of the obstacles that come from the inertia and enforced consistency of large systems are reduced dramatically in software systems that are built from small, largely independent programs that interface through files. Each program can use data structures, memory management and event management suited only for one task. The result can be smaller programs that are easier and faster to create and maintain and which can be re-engineered quickly when the inevitable knowledge that comes with widespread use of software in real-world applications motivates a major reorganization of the program. From the user perspective, because the results of each step in such a pipeline are captured as files, it is also easier to create or use additional third-party programs such as Matlab (The Mathworks, Inc.), OsiriX (http://www.osirix-viewer.com) or even Photoshop (Adobe Systems, Inc.) to perform customized manipulations or analyses, further adding to the flexibility of the overall system. With some expertise, it is also possible to gather such a set of individual programs into a scriptable, and thus automated, workflow. BioMesh3D is just such a system, driven by scripts written in Python that call individual programs, pass the data files and provide interactive control and feedback to the user.

In summary, image-based modelling and simulation appear to be paradigms with growing implications and opportunities for biomedical research and software systems to support the resulting pipeline that are bound to continue to grow and become more robust and useful for biomedical scientists. The question of which software architecture offers the most effective infrastructure for the development of such systems remains open. We have shown biomedical examples using both strategies and will continue to explore the benefits of both, driven always by the close collaborations with biomedical scientists that motivate the development and ultimately determine its success.
